# Investigation of Genetic Alterations Associated With Interval Breast Cancer

**DOI:** 10.1001/jamaoncol.2023.6287

**Published:** 2024-01-25

**Authors:** Juan Rodriguez, Felix Grassmann, Qingyang Xiao, Mikael Eriksson, Xinhe Mao, Svetlana Bajalica-Lagercrantz, Per Hall, Kamila Czene

**Affiliations:** 1 Department of Medical Epidemiology and Biostatistics, Karolinska Institutet, Stockholm, Sweden; 2 Health and Medical University, Potsdam, Germany; 3 Department of Oncology-Pathology, Karolinska Universitetssjukhuset, Stockholm, Sweden; 4 Department of Oncology, Södersjukhuset, Stockholm, Sweden

## Abstract

**Importance:**

Breast cancers (BCs) diagnosed between 2 screening examinations are called interval cancers (ICs), and they have worse clinicopathological characteristics and poorer prognosis than screen-detected cancers (SDCs). However, the association of rare germline genetic variants with IC have not been studied.

**Objective:**

To evaluate whether rare germline deleterious protein-truncating variants (PTVs) can be applied to discriminate between IC and SDC while considering mammographic density.

**Design, Setting, and Participants:**

This population-based genetic association study was based on women aged 40 to 76 years who were attending mammographic screening in Sweden. All women with a diagnosis of BC between January 2001 and January 2016 were included, together with age-matched controls. Patients with BC were followed up for survival until 2021. Statistical analysis was performed from September 2021 to December 2022.

**Exposure:**

Germline PTVs in 34 BC susceptibility genes as analyzed by targeted sequencing.

**Main Outcomes and Measures:**

Odds ratios (ORs) were used to compare IC with SDC using logistic regression. Hazard ratios were used to investigate BC-specific survival using Cox regression.

**Results:**

All 4121 patients with BC (IC, n = 1229; SDC, n = 2892) were female, with a mean (SD) age of 55.5 (7.1) years. There were 5631 age-matched controls. The PTVs of the *ATM*, *BRCA1*, *BRCA2*, *CHEK2*, and *PALB2* genes were more common in patients with IC compared with SDC (OR, 1.48; 95% CI, 1.06-2.05). This association was primarily influenced by *BRCA1/2 *and *PALB2* variants. A family history of BC together with PTVs of any of these genes synergistically increased the probability of receiving a diagnosis of IC rather than SDC (OR, 3.95; 95% CI, 1.97-7.92). Furthermore, 10-year BC-specific survival revealed that if a patient received a diagnosis of an IC, carriers of PTVs in any of these 5 genes had significantly worse survival compared with patients not carrying any of them (hazard ratio, 2.04; 95% CI, 1.06-3.92). All of these associations were further pronounced in a subset of patients with IC who had a low mammographic density at prior screening examination.

**Conclusions and Relevance:**

The results of this study may be helpful in future optimizations of screening programs that aim to lower mortality as well as the clinical treatment of patients with BC.

## Key Points

**Question:**

Can rare germline genetic variants be used to discriminate between interval and screen-detected breast cancer?

**Findings:**

In this genetic association study of 4121 patients with cancer and 5631 healthy controls, protein-truncating variants in the 5 major genes for breast cancer, particularly *BRCA1/2* and *PALB2* variants, were significantly associated with the occurrence of interval cancer. Women with a diagnosis of interval cancer who were carrying variants in any of these 5 genes experienced lower survival rates compared with women with interval cancer without such variants.

**Meaning:**

The results of this study suggest that screen-detected and interval cancers possess distinct genetic profiles; consequently, these findings may provide insights into the identification of individuals at high risk of developing aggressive forms of breast cancer.

## Teaser

This genetic association study examines whether rare germline deleterious protein-truncating variants can be applied to discriminate between interval cancer and screen-detected cancer while considering mammographic density in women with breast cancer.

## Introduction

Although breast cancer (BC) incidence has increased during the last decades, the mortality has decreased over the same period.^1^ One of the main reasons for the improved survival is the establishment of mammographic screening programs in many high-income countries.^2^ However, approximately 30% of BCs are not detected at screening^3^ but rather between 2 screening examinations, and are known as interval BC (IC).

ICs are generally diagnosed at later stages, and the patients are more likely to present metastases in local lymph nodes than patients with a diagnosis of screen-detected cancer (SDC).^4,5^ In addition, ICs are more likely to be estrogen receptor (ER)–negative, progesterone receptor (PR)–negative,^6,7^ and triple-negative^8^ compared with SDC. Thus, ICs have been claimed to be associated with a worse prognosis.^9^ Family history of BC within first-degree relatives has been associated with an increased risk of IC,^10,11,12,13^ and those patients with IC are also more likely than those with SDC to develop other tumors than BC, indicating a shared genetic cause.^14^

Higher mammographic density, quantified as percentage density (PD), reduces mammographic screening sensitivity, thereby increasing the likelihood of a subsequent IC, a phenomenon named masking.^8^ An IC could either be missed or masked at a prior screening, or be a fast-growing tumor. Missed or masked tumors are not considered true IC in contrast to a tumor that was not present at a prior screen. Therefore, the availability of mammographic density data makes it possible to study the determinants associated with true IC.

Genetic determinants of BC have been analyzed in a case-control meta-analysis, including more than 113 000 women from the Breast Cancer Association Consortium (BCAC).^15^ A total of 34 known or suspected BC susceptibility genes were investigated. Germline protein-truncating variants (PTVs) in 5 genes (*ATM*, *BRCA1*, *BRCA2*, *CHEK2*, and *PALB2*) were strongly associated with a risk of BC.^15^ PTVs in these 5 genes (hereinafter referred to as the 5 major genes for BC) are used for providing future cancer risks in the multifactorial Breast and Ovarian Analysis of Disease Incidence and Carrier Estimation Algorithm (BOADICEA) model^16,17^ included in the CanRisk tool.^18^ However, to our knowledge, the specific association of these genes with IC or SDC has not been studied.

In this article, we aim to identify germline deleterious PTVs associated with IC rather than SDC while considering mammographic density. Furthermore, we aim to investigate if these PTVs are associated with a worse BC-specific survival among patients with IC.

## Methods

### Study Population

The study population included women from Swedish cohorts Karolinska Mammography Project for Risk Prediction of Breast Cancer (KARMA) and prevalent KARMA (pKARMA). pKARMA consists of BC cases diagnosed in Stockholm between 2001 and 2008.^8,19^ KARMA is a large and well-characterized prospective screening cohort derived from population-based mammographic-screening or clinical radiology examinations conducted at 4 Swedish hospitals.^20^ BCs diagnosed without a prior screening visit or after a normal screening interval had passed (n = 2938) were excluded from the analyses as our main interest was ICs and SDCs. The final analytical data set combining pKARMA and KARMA comprised 9752 samples (1229 individuals with IC, 2892 individuals with SDC, and 5631 healthy controls; see eFigure 1 in Supplement 1  for a flowchart of the study creation). Basic information about SDC and IC cases from pKARMA and KARMA cohorts showed no differences in age at diagnosis, percentage mammographic density, or menopausal status, among others (eTable 1 in Supplement 1). All women included in the analyses gave written informed consent to the retrieval of data from medical records and national registers, answered a detailed questionnaire on background and lifestyle risk factors, and provided a blood specimen for genetic analysis. Ethical approvals were given by the ethical review board at Karolinska Institutet (Stockholm, Sweden). For more detailed information on the profile from both cohorts, we refer to our previous publications.^14,21,22,23^

### IC Ascertainment

The Stockholm mammography screening program started in 1989, and currently all women aged 40 to 74 years are invited to participate every 2 years. The participation rate for mammography screening in this program is high, exceeding 70%. Full details of the organizational and quality aspects of the program have been previously described.^24^

All patients with BC were assessed for screening history using dates of mammographic screening visits and information about the outcome, as described previously.^8,14^ In short, cancers diagnosed at a regularly scheduled mammographic screening were deemed SDC. Conversely, IC was defined as a BC diagnosis made after a negative screen but before the next visit. Women who did not attend the screening mammogram before BC diagnosis or with missing prior screening information were excluded from our analyses.

### Assessment of Mammographic Density

Mammographic density was measured in the prior screening mammogram (ie, the last negative mammogram preceding BC diagnosis) using STRATUS.^25^ Two different approaches were used for the comparison between patients with IC and SDC. The first approach included the entire data set adjusting for PD (based on quartiles). The second approach used a subset of IC cases with low PD, excluding patients with a diagnosis of IC (but not SDC) with medium or high mammographic density (PD, >25). This second approach has been previously used in our group^14^ to identify true IC cases not diluted by missed or masked tumors.

### Ascertainment of Genetic Profile and Family History

For retrieving PTV information, blood samples were sequenced at the Centre for Cancer Genetic Epidemiology (University of Cambridge; Cambridge, England). The 34 known or suspected risk gene panels were sequenced using the Hiseq4000 platform, which covered coding region and exon-intron boundaries. The workflow of sequencing experiments, bioinformatic analyses, and the definition of PTVs can be found in the eMethods in mentioned BCAC study, for which the KARMA cohort was part of the collaborative effort.^15^ In short, PTVs were defined as variants that disrupt gene function by introducing a stop codon (nonsense mutation), by frameshift insertion/deletions, or through splice site mutations. PTV carriership was defined as having at least 1 PTV in 1 of the 34 BC genes and was analyzed as a binary exposure (13 patients with BC and 7 controls were carriers of 2 PTVs, and they were considered as carriers of 1 PTV, contributing only in the highest-risk category of the 2 PTVs). The frequency of the 34 predisposition PTVs among all BC cases included in our data set is shown in eFigure 2 and eTable 2 in the Supplement 1.

For retrieving information on family history of BC, we used data from the Swedish Multi-Generation Register.^26^ For 523 patients (13%) with missing information, questionnaires answered by the participants were used. A positive family history of BC was defined as having a mother and/or sister(s) with BC.

### Tumor Characteristics, Treatment, and Survival

Tumor characteristics were retrieved from the National Quality Register for Breast Cancer.^27^ Basic adjustments for survival analyses included age at diagnosis (<50, 50–59, ≥60 years), year of diagnosis (2001-2004, 2005-2008, and 2008 and onwards), study cohort, and PD. Tumor characteristics adjustments included ER status, PR status, *ERBB2 *(formerly *HER2*) status, lymph node involvement, grade (well-differentiated, moderately differentiated, poorly differentiated), and tumor size (<20 mm, ≥20 mm). Treatment regimen adjustments included adjuvant chemotherapy, adjuvant hormone therapy, and radiotherapy. Date and cause of death was obtained through matching to the Swedish Cause of Death Register, with virtually no missing data (linkage performed on March 15, 2021).

### Statistical Analyses

All statistical analyses were performed in R, version 4.2.0 (R Foundation). Statistical significance was set at *P *< .05. We used a logistic regression to identify factors that were significantly different between patients with BC and controls and between IC and SDC.

Multivariable Cox proportional hazard regression models were used to estimate hazard ratios (HRs) for 10-year BC-specific survival from R package “survival,” with time since diagnosis as the underlying time scale. Time at risk was calculated from the date of study entry (ie, blood draw, left truncation) until the date of death due to BC, date of death of any cause, or end of follow-up, whichever came first. Kaplan-Meier plots (univariate, log-rank test) were visualized using the ggsurvplot function in the R package “survminer.”

## Results

### Investigating PTVs in BC Susceptibility Genes

Compared with controls, patients with BC were more likely to carry PTVs in any of the 34 BC genes. PTVs in the 5 major genes for BC (*ATM*, *BRCA1*, *BRCA2*, *CHEK2*, and *PALB2*), were all more often found among patients with BC than among controls (Table 1), and the associated values aligned with the ones reported in the meta-analysis from BCAC.^15^

**Table 1.** Association of PTVs With BC Risk, as Stratified by Mode of Detection

PTVs	BC vs controls, adjusted by age at baseline (n = 9752)^a^			SDC vs controls, adjusted by age at baseline (n = 8523)^b^			IC vs controls, adjusted by age at baseline (n = 6860)^c^		
	No.		BC vs controls, OR (95% CI)	No.		SDC vs controls, OR (95% CI)	No.		IC vs controls, OR (95% CI)
	Controls	BC		Controls	SDC		Controls	IC	
**Full panel (34 PTVs)**									
Noncarriers	5422	3819	1 [Reference]	5422	2686	1 [Reference]	5422	1133	1 [Reference]
Carriers	209	302	2.04 (1.70-2.44)	209	206	1.99 (1.63-2.42)	209	96	2.17 (1.69-2.79)
**Panel excluding 5 major genes**									
Noncarriers	5422	3819	1 [Reference]	5422	2686	1 [Reference]	5422	1133	1 [Reference]
Carriers	120	134	1.58 (1.23–2.03)	120	101	1.70 (1.30-2.22)	120	33	1.31 (0.88-1.93)
**5 Major genes for BC**									
Noncarriers	5542	3953	1 [Reference]	5542	2787	1 [Reference]	5542	1166	1 [Reference]
Carriers	89	168	2.62 (2.02-3.40)	89	105	2.34 (1.76-3.11)	89	63	3.30 (2.38-4.59)
***BRCA1/2* + *PALB2***									
Noncarriers	5542	3953	1 [Reference]	5422	2787	1 [Reference]	5422	1166	1 [Reference]
Carriers	18	68	5.24 (3.11-8.83)	18	37	4.07 (2.31-7.17)	18	31	7.99 (4.45-14.35)
***ATM* + *CHEK2***									
Noncarriers	5542	3953	1 [Reference]	5422	2787	1 [Reference]	5422	1166	1 [Reference]
Carriers	71	101	1.98 (1.46-2.69)	71	68	1.90 (1.36-2.66)	71	33	2.17 (1.43-3.30)
***BRCA1***									
Noncarriers	5542	3953	1 [Reference]	5542	2787	1 [Reference]	5542	1166	1 [Reference]
Carriers	3	12	5.59 (1.58-19.83)	3	6	3.97 (0.99-15.89)	3	6	9.54 (2.38-38.24)
***BRCA2***									
Noncarriers	5542	3953	1 [Reference]	5542	2787	1 [Reference]	5542	1166	1 [Reference]
Carriers	10	42	5.81 (2.91-11.60)	10	23	4.56 (2.16-9.59)	10	19	8.74 (4.05-18.85)
***PALB2***									
Noncarriers	5542	3953	1 [Reference]	5542	2787	1 [Reference]	5542	1166	1 [Reference]
Carriers	5	14	3.89 (1.40-10.80)	5	8	3.17 (1.04-9.70)	5	6	5.59 (1.70-18.36)
***ATM***									
Noncarriers	5542	3953	1 [Reference]	5542	2787	1 [Reference]	5542	1166	1 [Reference]
Carriers	12	27	3.15 (1.59-6.22)	12	19	3.14 (1.52-6.48)	12	8	3.18 (1.29-7.79)
***CHEK2***									
Noncarriers	5542	3953	1 [Reference]	5542	2787	1 [Reference]	5542	1166	1 [Reference]
Carriers	59	74	1.74 (1.23-2.46)	59	49	1.65 (1.12-2.41)	59	25	1.97 (1.23-3.16)

Abbreviations: BC, breast cancer; IC, interval breast cancer; OR, odds ratio; PTVs, protein-truncating variants; SDC, screen-detected breast cancer.

^a^ We included patients with BC with a diagnosis of SDC (n = 2892) and IC (n = 1229) and compared them with healthy controls (n = 5631). PTVs are grouped into different panels (based on the previously reported association with BC), followed by individualized analysis for the 5 major genes for BC. The left column includes all patients with BC attending to mammographic screening (SDC and IC) compared with controls.

^b^ The middle column includes patients with a diagnosis of SDC compared with controls.

^c^ The right column includes patients with a diagnosis of IC compared with controls. All models were adjusted by age at baseline.

### Association of PTVs in the 5 Major Genes With Risk of IC and SDC

Next, we investigated the association with PTVs in the 5 major genes for BC, directly comparing 1229 patients with a diagnosis of IC with 2892 patients with SDC. Patients with IC were more likely to carry PTVs in the 5 major genes (odds ratio [OR], 1.48; 95% CI, 1.06-2.05) compared with SDC (Table 2). This finding was further pronounced in a subset of IC with breasts of low mammographic density (OR, 1.87; 95% CI, 1.22-2.85), in which only true IC was considered (those cancers with PD <25% and therefore with lower probability of being missed at the previous screening). Using a high-dense subset for IC (PD, ≥25%) did not show a significant difference compared with SDC (OR, 1.24; 95% CI, 0.63-1.70).

**Table 2.** Association of PTVs With IC

PTVs	All patients with BC, adjusted for age at diagnosis, study cohort, and PD (n = 4121)^a^			True IC subset, adjusted for age at diagnosis and study cohort (n = 3377)^b^		
	No.		IC vs SDC, OR (95% CI)	No.		IC vs SDC, OR (95% CI)
	SDC	IC		SDC	IC	
**Full panel (34 PTVs)**						
Noncarriers	2686	1133	1 [Reference]	2686	437	1 [Reference]
Carriers	206	96	1.14 (0.88-1.47)	206	48	1.47 (1.05-2.05)
**All PTVs excluding 5 major genes**						
Noncarriers	2686	1133	1 [Reference]	2686	437	1 [Reference]
Carriers	101	33	0.80 (0.53-1.20)	101	18	1.07 (0.64-1.80)
**5 major genes for BC**						
Noncarriers	2787	1166	1 [Reference]	2787	455	1 [Reference]
Carriers	105	63	1.48 (1.06-2.05)	105	30	1.87 (1.22-2.85)
***BRCA1/2* + *PALB2***						
Noncarriers	2787	1166	1 [Reference]	2787	455	1 [Reference]
Carriers	37	31	1.92 (1.17-3.15)	37	15	2.54 (1.37-4.69)
***ATM* + *CHEK2***						
Noncarriers	2787	1166	1 [Reference]	2787	455	1 [Reference]
Carriers	68	33	1.24 (0.80-1.92)	68	15	1.48 (0.83-2.62)

Abbreviations: BC, breast cancer; IC, interval breast cancer, OR, odds ratio, PD, percentage density; PTVs, protein-truncating variants; SDC, screen-detected breast cancer.

^a^ We included patients with BC patients with SDC (n = 2892) and IC (n = 1229). PTVs are grouped into different panels based on the previously reported significant association with BC. The left column included all patients with BC attending to mammographic screening (SDC and IC), adjusted by age at diagnosis, study cohort, and PD.

^b^ Patients with IC (but not SDC) with medium or high mammographic density (PD, >25) were excluded. See eTable 5 in Supplement 1 for individualized analysis for each PTV.

When stratifying the 5 major genes panel into high risk (*BRCA1/2* + *PALB2*) and moderate-high risk (*ATM* + *CHEK2*) for BC, we found that patients with IC were more likely than those with SDC to carry PTVs for *BRCA1*/*2* + *PALB2* (Table 2). These associations were also seen in women without a strong family history of BC (defined as 2 or more family members with breast or ovarian cancer, or 1 family member with breast and 1 with ovarian cancer; eTable 3 in Supplement 1).

### Association of Family History of BC in Combination With PTVs in the 5 Major Genes With Risk for Developing IC

Having a first-degree family member with BC has previously been found to be associated with an increased risk of IC.^10,11,12,13^ In our data set, relatives with BC were more often found among patients with IC than among those with SDC (OR, 1.25; 95% CI, 1.05-1.48). Interaction analyses showed that the association between PTVs in the 5 major BC genes and risk of IC vs SDC was statistically higher among women with family history of BC, with ORs of 3.95 (95% CI, 1.97-7.92) and 5.17 (95% CI, 2.26-11.85) for the entire data set and true IC subset, respectively (Table 3).

**Table 3.** Association of Combined Association of PTVs in the 5 Major Genes and FH-BC With IC

FH-BC and PTVs in the 5 major genes for BC status	All patients with BC, adjusted for age at diagnosis, study cohort, No. of relatives, and PD (n = 4121)^a^			True IC subset, adjusted for age at diagnosis, No. of relatives, and study cohort (n = 3377)^b^		
	No.		IC vs SDC, OR (95% CI)	No.		IC vs SDC, OR (95% CI)
	SDC	IC		SDC	IC	
No FH-BC and no PTVs	2268	930	1 [Reference]	2268	354	1 [Reference]
FH-BC and no PTVs	489	229	1.17 (0.98-1.41)	489	97	1.29 (1.01-1.66)
No FH-BC and PTVs	89	40	1.13 (0.76-1.67)	89	20	1.53 (0.93-2.53)
FH-BC and PTVs	14	22	3.95 (1.97-7.92)	14	10	5.17 (2.26-11.85)
Interaction FH-BC PTVs	NA	NA	2.98 (1.33-6.69)	NA	NA	2.62 (0.98-7.02)
*P* value	NA	NA	.01	NA	NA	.06

Abbreviations: BC, breast cancer; FH-BC, family history of breast cancer; IC, interval breast cancer; NA, not applicable; OR, odds ratio, PD, percentage density; PTVs, protein-truncating variants; SDC, screen-detected breast cancer.

^a^ We included patients with BC with SDC (n = 2892) and IC (n = 1229), stratified according to the status of FH-BC and PTVs in the 5 major genes for BC. We divided the data set into 4 different subgroups based on the absence of FH- BC and absence of PTVs in the 5 major genes (first group, reference), the presence of 1 of these factors (second and third groups), or the concurrence of these 2 factors (fourth group). The left column included all patients with BC attending to mammographic screening (SDC and IC), adjusted by age at diagnosis, study cohort, number of female relatives, and PD.

^b^ Patients with IC (but not SDC) with medium or high mammographic density (PD, >25) were excluded. The statistical interaction between the 2 binary exposures (FH-BC and PTVs in the 5 major genes) is reported. 32 SDC and 8 IC (4 “true” IC) cases had missing information regarding FH-BC.

### Association of PTVs in the 5 Major Genes With Worse Survival Among Women With IC

After excluding 488 in situ BC cases, the prognosis of the remaining 3633 women with BC participating in mammographic screening was investigated. Tumor characteristics of the 3633 patients with BC included in this analysis are described in eTable 4 in Supplement 1. In Cox regression models, carriers of PTVs in any of the 5 major genes were associated with worse 10-year BC-specific mortality than noncarriers (HR, 1.89; 95% CI, 1.08-3.30). Importantly, the significant association between PTV carriership and worse survival remained in the subset comprising the 1154 women with a diagnosis of IC (Figure and Table 4). The fully adjusted model (including tumor characteristics and treatment) showed an association of PTVs in the 5 major genes with worse survival when considering all IC (HR, 2.04; 95% CI, 1.06-3.92) and true IC tumors (HR, 2.97; 95% CI, 1.07-8.25; Table 4), respectively.

**Figure.**
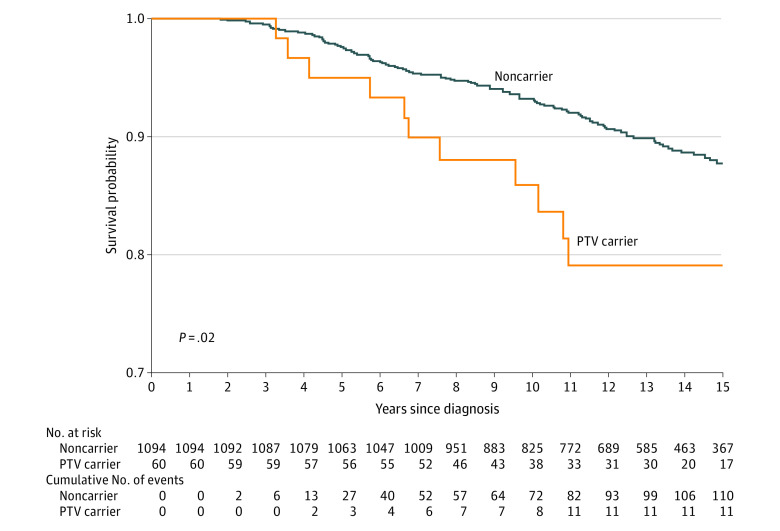
Breast Cancer (BC)–Specific Survival in Patients With Interval Cancer (IC) Kaplan-Meier plot and log-rank test showing 15-year BC-specific survival in 1154 patients with IC. In situ cancers (n = 75) were excluded. Patients have been stratified into carriers (orange line) and noncarriers (blue line) of protein-truncating variants (PTVs) in any of the 5 major genes for BC (*BRCA1*, *BRCA2*, *PALB2*, *ATM*, and *CHEK2*).

**Table 4.** Association of BC-Specific Survival With PTVs in the 5 Major Genes, as Stratified by Mode of Detection

Patients with BC	No. of patients	HR (95% CI)		
		Adjusted for age at diagnosis, year of diagnosis, PD, and study cohort	Tumor characteristics	Treatment
All patients with BC	3633	2.24 (1.29-3.88)	1.98 (1.14-3.44)	1.89 (1.08-3.30)
SDC subset	2479	1.22 (0.38-3.92)	1.16 (0.36-3.76)	1.21 (0.37-3.91)
IC subset	1154	2.65 (1.40-5.00)	2.13 (1.12-4.06)	2.04 (1.06-3.92)
True IC subset^a^	456	2.07 (0.81-5.33)	2.73 (1.02-7.35)	2.97 (1.07-8.25)

Abbreviations: BC, breast cancer; HR, hazard ratio; IC, interval breast cancer; PD, percentage density; PTVs, protein-truncating variants; SDC, screen-detected breast cancer.

^a^ Patients were stratified based on mode of detection (IC or SDC) and right truncated at 10 years. “True IC subset” refers to the subset of patients who received a diagnosis of IC after exclusion of those with medium or high mammographic density (PD, >25).

## Discussion

In this study, we provided 2 clinical take-home messages. First, we showed that germline PTVs in *ATM*, *BRCA1*, *BRCA2*, *CHEK2*, and *PALB2* were associated with an increased risk of IC, and were mostly associated with *BRCA1*, *BRCA2*, and *PALB2*. Women with a family history of BC, in combination with deleterious variants in any of these 5 genes, were 4 times more likely to develop IC compared with SDC. Second, if a patient received a diagnosis of an IC, carriers of PTVs in any of these 5 genes had a significantly worse survival compared with women not carrying any of the pathogenic variants.

It has been shown that PTVs in 5 of the 34 putative BC genes are strongly associated with risk of BC, placing *PALB2* at the highest risk category together with *BRCA1/2*, and PTVs in *ATM* and *CHEK2* in the moderate–high-risk category.^15^ Our results aligned with these findings; PTVs in the 5 major BC genes were significantly more common in patients compared with controls. When directly comparing IC vs SDC subsets, we found that PTVs in *BRCA1/2* + *PALB2* genes conferred a 2-fold increased risk for IC compared with SDC. The 2 remaining major genes combined (*ATM* + *CHEK2*) were not statistically associated with IC. These results suggest that SDC and IC are distinct entities in underlying genetics and biology. To our knowledge, this is the first report looking into the genetic differences between SDC and IC using the 5 major genes, potentially making advances in our understanding of genetic susceptibility to these distinct breast cancers. We believe that genetic risk discrimination has potential applicability in clinical care, since women with an IC currently do not benefit from BC screening.

PTVs of the 5 major genes for BC are included in the BOADICEA model for estimating the future risks of developing breast or ovarian cancer, together with information on cancer family history and common genetic variants.^16,17,28,29,30^ A family history of BC has previously been found to be associated with an increased risk of IC.^13^ The results of the current study suggest that patients with PTVs in any of the 5 major genes and family history of BC have a 4-fold to 5-fold increased risk of receiving a diagnosis of IC compared with SDC. The increase was approximately 3 times higher than the expected additive effect of both factors, suggesting unidentified genetic determinants associated with IC, thus providing potentially valuable information for identifying women who are at a very high risk for developing an aggressive BC.

In this study, we offered insights into the genetic underpinnings of the adverse prognostic features associated with IC.^8,31^ The results found that carriers of PTVs in any of the 5 major genes displayed a higher mortality among women with IC (approximately 2-fold to 3-fold increased mortality) compared with noncarriers, even after adjusting for known tumor characteristics and treatment. Deficiency in the homologous recombination repair pathway, an accurate, error-free DNA repair mechanism in which all of the 5 major genes have a key role, could potentially be associated with accelerated acquisition of mutations beneficial for tumor growth and survival,^32,33,34^ thus resulting in a worse prognosis for those carrying such variants. The association of the PTVs in those genes with IC aligns with the observation that carriers of those mutations are at risk for more aggressive, higher-grade tumors.^35^ The elevated tumor grade may be associated with accelerated tumor progression, prompting self-detection by the patient during the intervals between screening rounds rather than at the subsequent screening appointment. Nevertheless, because these mutations are rare, they can explain only a small proportion of IC cases. Most affected women likely possess different, presently unidentified risk factors, whether genetic or nongenetic in nature.

Mammographic screening protocols in Sweden have remained unchanged for decades. Special screening regimens exist for women with *BRCA1/2* variants or a strong family history of breast/ovarian cancer.^36,37^ These regimens include earlier screening, shorter intervals, and additional imaging methods. Genetic counseling is shifting toward broader gene panels. Our study found that *PALB2* variants, along with *BRCA1/2* variants, were associated with increased breast cancer risk. This emphasizes the need for more frequent screening for these women, aligning with recent European guidelines.^38^

While our findings might be relevant for genetic counseling of high-risk families, we foresee the main implication for the general population of women attending population-based mammography screening programs. Implementation of personalized risk-based screening programs is foreseeable in the near future. Our study demonstrates that variant testing in healthy women attending screening might help to identify women at high risk to develop IC. In addition, the diminishing cost of variant testing can provide a high health care value of testing all patients with a new diagnosis of IC to identify those with bad prognosis.

### Strengths and Limitations

A potential limitation of our study could be the awareness of being a *BRCA1/2* carrier and the resulting participation in special screening regimens. However, only 2 patients with BC in our study were aware of carrying a *BRCA1/2* variant, which represents a small percentage (3.7%) and is unlikely to have affected our results. Sensitivity analyses of the association between *BRCA1/2* (and *PALB2*) variants and breast cancer in women without a strong family history yielded consistent estimates. One weakness is the limited statistical power for prognosis analyses, especially among patients with SDC, due to a small number of events. In the context of mammography screening investigations, this study finds optimal relevance within countries whose screening programs align with the framework in Sweden. Extrapolating to countries with different screening regimens necessitates further investigation. The study’s major strength lies in the extensive national data available, representing a large sample of the Swedish population and sequenced women. It provides a platform to understand molecular patterns in BC. Additionally, our study considered mammographic density, allowing us to identify aggressive interval tumors that were not missed during screenings but emerged between screens.

## Conclusions

The results of this genetic association study suggest that women who inherited PTVs of the 5 major genes for BC disproportionately contributed to the diagnosis of IC, and it was mostly associated with *BRCA1/2* and *PALB2* genes. The synergistic effect of family history of BC and the 5 major genes suggests that further large-scale sequencing efforts are necessary to uncover the full genetic contribution of the observed interaction. Our results also suggested that PTVs in those 5 major genes are associated with a worse survival among patients with IC. Our work potentially clarifies the picture of what type of BC is likely to evade detection in population-based screening programs. These insights will possibly be helpful in future optimizations of screening programs aimed at lowering mortality, as well as the clinical treatment of patients with BC.

## Notes

Supplement 1. eTable 1. Baseline characteristics of BC patients, stratified by study cohort eTable 2. Frequency of germline protein-truncating variants in the 34 BC genes eTable 3. Association of protein-truncating variants in the 5 major genes with interval cancer among women without strong family history of BC eTable 4. Tumor characteristics of BC patients, stratified by mode of detection eTable 5. Association of protein-truncating variants in the 5 major genes with interval cancer eFigure 1. CONSORT flow diagram eFigure 2. Frequency of Protein-Truncating Variants among BC patients

Supplement 2. Data sharing statement
