# Supplementary material for: Investigation of Genetic Alterations Associated With Interval Breast Cancer
Source: JAMA Oncol. 2024 Jan 25;10(3):372–9. doi: 10.1001/jamaoncol.2023.6287 (PMC10811589; doi:10.1001/jamaoncol.2023.6287)
Supplement: Supplement 1. — eTable 1. Baseline characteristics of BC patients, stratified by study cohort eTable 2. Frequency of germline protein-truncating variants in the 34 BC genes eTable 3. Association of protein-truncating variants in the 5 major genes with interval cancer among women without strong family history of BC eTable 4. Tumor characteristics of BC patients, stratified by mode of detection eTable 5. Association of protein-truncating variants in the 5 major genes with interval cancer eFigure 1. CONSORT flow diagram eFigure 2. Frequency of Protein-Truncating Variants among BC patients [file jamaoncol-e236287-s001.pdf]

Supplemental Online Content

Rodriguez J, Grassmann F, Xiao Q, et al. Investigation of genetic alterations associated with interval breast cancer. *JAMA Oncol*. Published online January 25, 2023.
doi:10.1001/jamaoncol.2023.6287

eTable 1. Baseline characteristics of BC patients, stratified by study cohort
eTable 2. Frequency of germline protein-truncating variants in the 34 BC genes
eTable 3. Association of protein-truncating variants in the 5 major genes with interval cancer among women without strong family history of BC
eTable 4. Tumor characteristics of BC patients, stratified by mode of detection
eTable 5. Association of protein-truncating variants in the 5 major genes with interval cancer
eFigure 1. CONSORT flow diagram
eFigure 2. Frequency of Protein-Truncating Variants among BC patients

This supplemental material has been provided by the authors to give readers additional information about their work.

© 2024 Rodriguez J et al. *JAMA Oncology.*

eTable 1. Baseline characteristics of BC patients, stratified by study cohort

Baseline characteristics	pKARMA<br>*n* = 2,471	KARMA<br>*n* = 1,650	pKARMA + KARMA<br>*n* = 4,121
Age at diagnosis in years, mean (SD)	59.4 (6.0)	59.6 (8.4)	59.5 (7.1)
Age at study entry in years, mean (SD)	59.4 (6.0)	60.6 (8.6)	59.9 (7.2)
Age at menarche in years, mean (SD)	13.2 (1.5)	13.2 (1.5)	13.2 (1.5)
Percent mammographic density (PD), mean (SD)	22 (13.3)	23.5 (18.7)	22.5 (15.5)
Body mass index in kg/m2, mean (SD)	25.4 (4.1)	25.9 (4.3)	25.6 (4.2)
Menopausal status at diagnosis, *n* (%)			
Premenopausal	232 (9.4)	261 (15.8)	493 (12.0)
Postmenopausal	2,239 (90.6)	1,389 (84.2)	3,628 (88.0)
Missing	0 (0)	0 (0)	0 (0)
Education, *n* (%)			
Elementary	378 (15.3)	226 (13.7)	604 (14.7)
Intermediate	546 (22.1)	545 (33.0)	1,091 (26.5)
University	1,043 (42.2)	726 (44.0)	1,769 (42.9)
Other/missing	504 (20.4)	153 (9.3)	657 (15.9)
Number of children, *n* (%)			
0	402 (16.3)	184 (11.2)	586 (14.2)
1	441 (17.9)	245 (14.8)	686 (16.6)
2	1,083 (43.8)	770 (46.7)	1,853 (45.0)
≥3	534 (21.6)	367 (22.2)	901 (21.9)
Missing	11 (0.4)	84 (5.1)	95 (2.3)

Baseline characteristics of all BC patients from pKARMA, KARMA, and both cohorts combined. NOTE: Significant associations ($P < 0.05$) are denoted in bold.

Abbreviation: *BC* breast cancer, *SD* standard deviation.

© 2024 Rodriguez J et al. *JAMA Oncology.*

eTable 2. Frequency of germline protein-truncating variants in the 34 BC genes

Gene name	Number of BC patients with a PTV	Frequency (%) in dataset (SDC & IC tumors)	Frequency (%) among PTVs (SDC & IC tumors)
CHEK2	74	1.80	23.49
FANCM	53	1.29	16.83
BRCA2	42	1.02	13.33
ATM	27	0.66	8.57
RAD50	15	0.36	4.76
PALB2	14	0.34	4.44
BRCA1	12	0.29	3.81
BARD1	10	0.24	3.17
NBN	8	0.19	2.54
RAD51C	8	0.19	2.54
BRIP1	7	0.17	2.22
ABRAXAS1	7	0.17	2.22
RECQL	7	0.17	2.22
FANCC	5	0.12	1.59
MUTYH	5	0.12	1.59
MRE11	4	0.10	1.27
STK11	3	0.07	0.95
BABAM2	2	0.05	0.63
GEN1	2	0.05	0.63
NF1	2	0.05	0.63
RINT1	2	0.05	0.63
AKT1	1	0.02	0.32
EPCAM	1	0.02	0.32
MSH6	1	0.02	0.32
PIK3CA	1	0.02	0.32
PTEN	1	0.02	0.32
XRCC2	1	0.02	0.32
CDH1	0	0.00	0.00
MEN1	0	0.00	0.00
MLH1	0	0.00	0.00
MSH2	0	0.00	0.00
PMS2	0	0.00	0.00
RAD51D	0	0.00	0.00
TP53	0	0.00	0.00

List of the 34 BC susceptibility genes, the frequency of PTVs in the dataset (2,892 SDC and 1,229 IC), and the relative frequency among the total number of PTVs in the dataset.

Abbreviation: *BC* breast cancer, *IC* interval breast cancer, *PTVs* protein-truncating variants, *SDC* screen-detected breast cancer.

© 2024 Rodriguez J et al. *JAMA Oncology.*

eTable 3. Association of protein-truncating variants in the 5 major genes with interval cancer among women without strong family history of BC

Protein-Truncating Variants (PTVs)	All BC patients Adj. for age at diagnosis, study cohort, and PD *N* = 4,005			'True' IC subset Adj. for age at diagnosis and study cohort *N* = 3,277		
	SDC (*N*)	IC (*N*)	IC vs. SDC OR (95% CI)	SDC (*N*)	IC (*N*)	IC vs. SDC OR (95% CI)
Full panel (34 PTVs)						
Non-Carriers	2,609	1,105	1.00 (Referent)	2,609	424	1.00 (Referent)
Carriers	197	94	1.16 (0.89–1.50)	197	47	**1.50 (1.07–2.10)**
All PTVs excl. 5 major genes						
Non-Carriers	2,609	1,105	1.00 (Referent)	2,609	424	1.00 (Referent)
Carriers	98	33	0.82 (0.54–1.23)	98	18	1.10 (0.66–1.85)
5 major genes for BC						
Non-Carriers	2,387	1,138	1.00 (Referent)	2,387	442	1.00 (Referent)
Carriers	99	61	**1.50 (1.07–2.10)**	99	29	**1.91 (1.25–2.94)**
BRCA1/2 + PALB2						
Non-Carriers	2,387	1,138	1.00 (Referent)	2,387	442	1.00 (Referent)
Carriers	34	29	**1.95 (1.16–3.26)**	34	14	**2.58 (1.36–4.87)**
ATM + CHEK2						
Non-Carriers	2,387	1,138	1.00 (Referent)	2,387	442	1.00 (Referent)
Carriers	65	33	1.28 (0.82–1.98)	65	15	1.54 (0.87–2.74)

We included BC patients diagnosed with SDC (=2,806) and IC (=1,199), after excluding 72 BC patients with strong family history of BC, and 44 BC patients with missing information. The left model included all BC patients attending to mammographic screening (SDC and IC), adjusted by age at diagnosis, study cohort and PD; In the right model "True IC subset", IC patients (but not SDC patients) with medium or high mammographic density (PD > 25) were excluded. Of note, the corresponding ORs for BRCA1/2 (without PALB2) were 2.03 (1.14–3.60) and 2.34 (1.11–4.91) for the entire dataset and 'true' IC subset, respectively (data not shown in tables). NOTE: Significant associations (*P* < 0.05) are denoted in bold.

Abbreviation: *BC* breast cancer, *CI* confidence interval, *IC* interval breast cancer, *OR* odds ratio, *PD* percent density *SDC* screen-detected breast cancer.

© 2024 Rodriguez J et al. *JAMA Oncology.*

eTable 4. Tumor characteristics of BC patients, stratified by mode of detection

Tumor characteristics	No. of patients diagnosed with SDC (%)	No. of patients diagnosed with IC (%)	Chi-square test P value
Year of diagnosis			0.69
2001–2004	705 (28.44%)	339 (29.37%)	
2005–2008	1,110 (44.78%)	497 (43.07%)	
2009–2012	472 (19.04%)	219 (18.98%)	
2013–2016	192 (7.74%)	99 (8.58%)	
Missing (0%)			
Age at diagnosis			**<0.001**
<50	151 (6.09%)	115 (9.96%)	
50–59	894 (36.06%)	473 (40.99%)	
≥60	1,434 (57.85%)	566 (49.05%)	
Missing (0%)			
ER status			**<0.001**
Positive	2,135 (90.62%)	875 (80.20%)	
Negative	221 (9.38%)	216 (19.80%)	
Missing (5.12%)			
PR status			**<0.001**
Positive	1,772 (75.66%)	689 (63.97%)	
Negative	570 (24.34%)	388 (36.03%)	
Missing (5.89%)			
HER2 status			**<0.001**
Positive	128 (10.47%)	107 (18.32%)	
Negative	1,095 (89.53%)	477 (81.68%)	
Missing (6.55%)			
Tumor size (mm)			**<0.001**
<20	1,771 (81.02%)	640 (63.05%)	
≥20	415 (18.98%)	375 (36.95%)	
Missing (11.89%)			
Lymph node involvement			**<0.001**
No	2,183 (93.41%)	906 (85.07%)	
Yes	154 (6.59%)	159 (14.93%)	
Missing (6.36%)			
Grade			**<0.001**
Well-differentiated	514 (26.62%)	133 (15.57%)	
Moderately differentiated	973 (50.39%)	393 (46.02%)	
Poorly differentiated	444 (22.99%)	328 (38.41%)	
Missing (4.39%)			

Tumor characteristics of the 3.633 BC patients attending to mammographic screening, after exclusion of in situ cancers (N=488). Patients were stratified by mode of detection (SDC or IC). Statistical differences between BC subtypes were calculated using the chi-square test. Missing values were excluded for calculating the proportions. HER2 status and grade were available in the registry for BC patients diagnosed from 2007 and 2004 onwards, respectively. NOTE: Significant associations ($P < 0.05$) are denoted in bold.

Abbreviation: *BC* breast cancer, *IC* interval breast cancer, *ER* estrogen receptor, *PR* progesterone receptor, *SDC* screen-detected breast cancer.

© 2024 Rodriguez J et al. *JAMA Oncology.*

eTable 5. Association of protein-truncating variants in the 5 major genes with interval cancer

Protein-Truncating Variants in the 5 major genes for BC	All BC patients Adj. for age at diagnosis, study cohort, and PD *n* = 4,121			'True' IC subset Adj. for age at diagnosis and study cohort *n* = 3,377		
	SDC (*n*)	IC (*n*)	IC vs. SDC OR (95% CI)	SDC (*n*)	IC (*n*)	IC vs. SDC OR (95% CI)
CHEK2						
Non-Carriers	2,787	1,166	1.00 (Referent)	2,787	455	1.00 (Referent)
Carriers	49	25	1.20 (0.73–1.99)	49	10	1.34 (0.67–2.68)
BRCA2						
Non-Carriers	2,787	1,166	1.00 (Referent)	2,787	455	1.00 (Referent)
Carriers	23	19	1.77 (0.95–3.32)	23	9	**2.40 (1.10–5.27)**
ATM						
Non-Carriers	2,787	1,166	1.00 (Referent)	2,787	455	1.00 (Referent)
Carriers	19	8	1.37 (0.58–3.23)	19	5	1.84 (0.68–4.99)
PALB2						
Non-Carriers	2,787	1,166	1.00 (Referent)	2,787	455	1.00 (Referent)
Carriers	8	6	1.99 (0.67–5.95)	8	5	**4.27 (1.37–13.32)**
BRCA1						
Non-Carriers	2,787	1,166	1.00 (Referent)	2,787	455	1.00 (Referent)
Carriers	6	6	2.42 (0.74–7.86)	6	1	0.99 (0.12–8.25)

We included BC patients diagnosed with SDC (=2,892) and IC (=1,229). The left model included all BC patients attending to mammographic screening (SDC and IC), adjusted by age at diagnosis, study cohort and PD; In the right model "True IC subset", IC patients (but not SDC patients) with medium or high mammographic density (PD > 25) were excluded. NOTE: Significant associations ($P < 0.05$) are denoted in bold.

Abbreviation: *BC* breast cancer, *CI* confidence interval, *IC* interval breast cancer, *OR* odds ratio, *PD* percent density *SDC* screen-detected breast cancer.

© 2024 Rodriguez J et al. *JAMA Oncology.*

Supplementary Figures

eFigure 1. CONSORT flow diagram

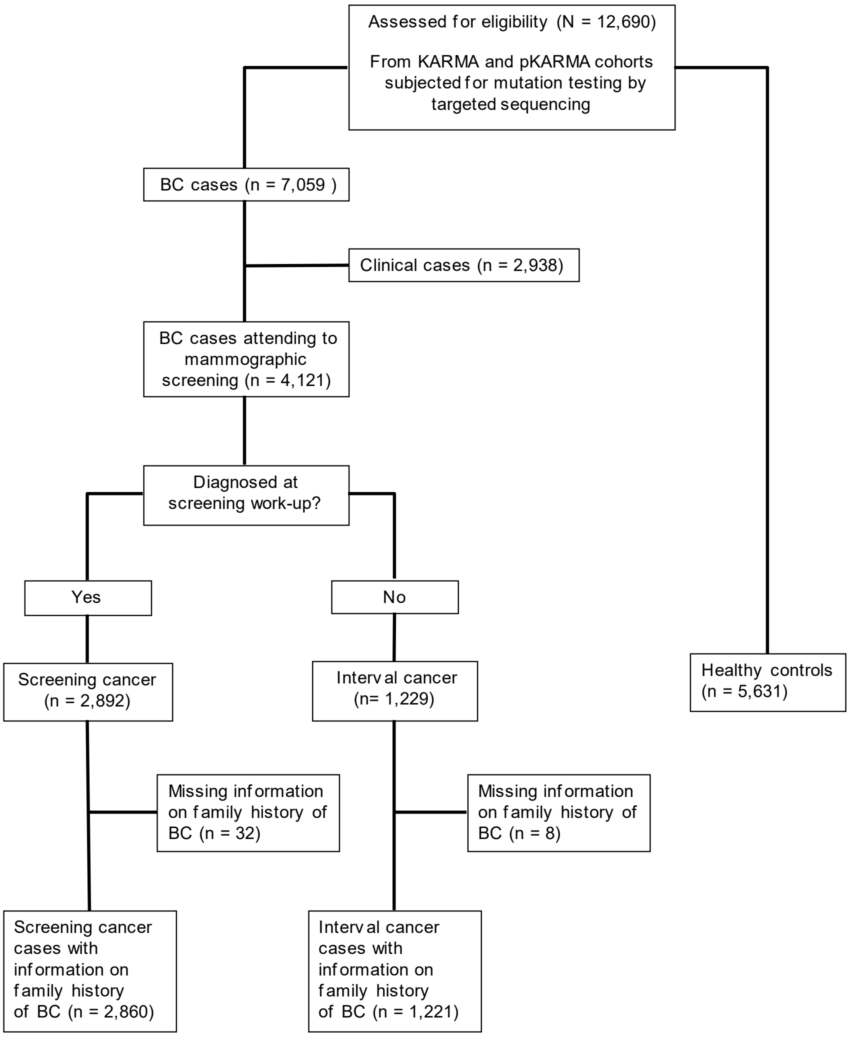

Flow chart of study creation, describing the initial cohort available for analysis and exclusions made. Only participants who were subjected for mutation testing using targeted sequencing and passed quality control were included. All BC cases from pKARMA, and all BC cases from KARMA diagnosed from 2005 to 2016 were included in the current study, together with age-matched controls. The first occurrence of BC was counted as the date of diagnosis. Clinical cancer cases (*N* = 2,938) were excluded from the analyses as our main interest was interval and screening detected cancers. 40 BC patients had missing information in both questionnaires and in The Swedish Multi-Generation Register, and that information was considered as missing (0.97% of the dataset).
Abbreviation: BC breast cancer.

© 2024 Rodriguez J et al. *JAMA Oncology.*

eFigure 2. Frequency of Protein-Truncating Variants among BC patients

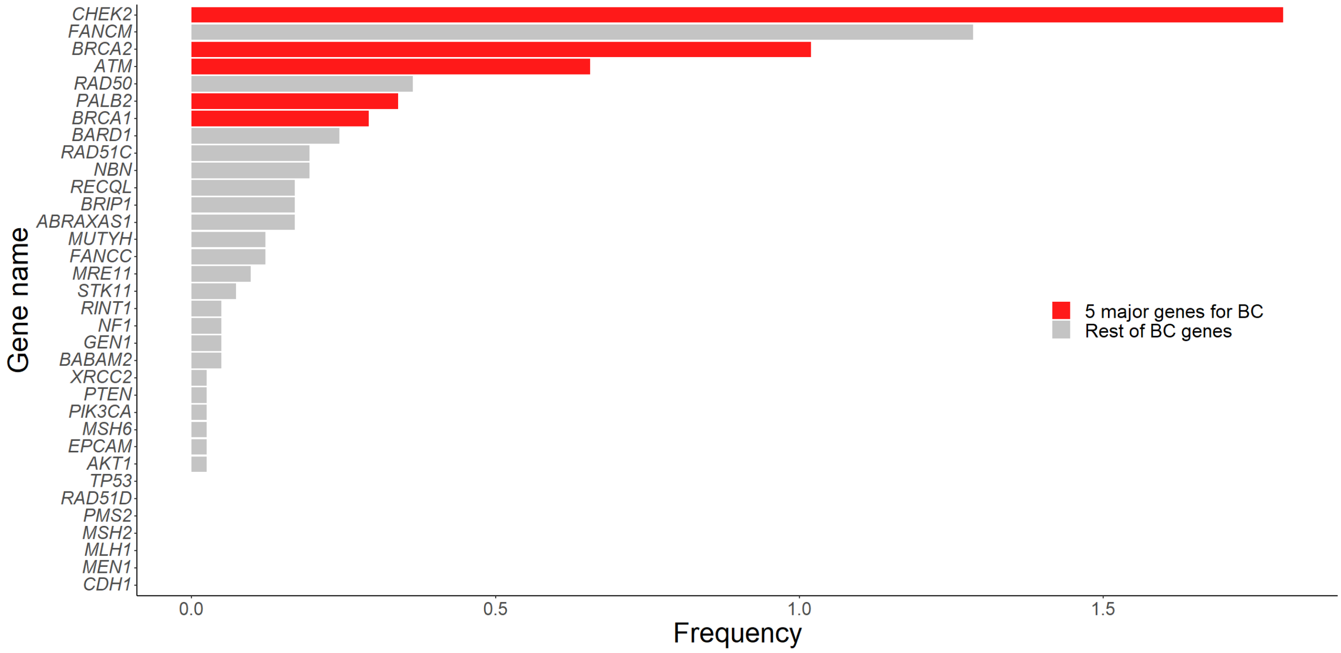

Frequency of PTVs in 34 susceptibility genes among 4,121 BC patients. PTVs were found in 96 (7,8%) patients with IC, and in 206 (7.1%) patients diagnosed with SDC. The genes are listed in order of decreasing frequency for BC patients. Red bars represent PTVs in the 5 major genes for BC, while grey bars represent PTVs in the 29 remaining genes. No PTV was found in *TP53*, *RAD51D*, *PMS2*, *MSH2*, *MLH1*, *MEN1*, and *CDH1* genes.

Abbreviation: *BC* breast cancer, *IC* interval breast cancer, *PTVs* protein-truncating variants, *SDC* screen-detected breast cancer.

© 2024 Rodriguez J et al. *JAMA Oncology*.
