# Supplementary material for: Investigation of Genetic Alterations Associated With Interval Breast Cancer
Source: JAMA Oncol. 2024 Jan 25;10(3):372–9. doi: 10.1001/jamaoncol.2023.6287 (PMC10811589; doi:10.1001/jamaoncol.2023.6287)
Supplement: Supplement 2. — Data sharing statement [file jamaoncol-e236287-s002.pdf]

Data Sharing Statement

Data

Data available: Yes
Data types: Participant data with identifiers
How to access data: Access to phenotypes, biospecimens and genotypes from the KARMA study can be requested from https://karmastudy.org/data-access/. Access to the pKARMA phenotypes and genotypes is restricted due to IRB requirements but data can be shared upon reasonable request to the principal investigator of pKARMA (Kamila.Czene@ki.se)
When available: With publication

Supporting Documents

Document types: Statistical/analytic code
How to access documents: juan.rodriguez@ki.se
When available: With publication

Additional Information

Who can access the data: Researchers whose proposed use of the data has been approved
Types of analyses: Any purpose
Mechanisms of data availability: After approval of a proposal
